# Prognostic significance of elevated pretreatment systemic inflammatory markers for patients with prostate cancer: a meta-analysis

**DOI:** 10.1186/s12935-019-0785-2

**Published:** 2019-03-25

**Authors:** Hao Peng, Xiaogang Luo

**Affiliations:** 1 Department of Urological Surgery, Zhoukou Central Hospital of Henan Province, No. 26 Renmin East Road, Chuanhui District, Zhoukou, 466000 China; 2 State Key Laboratory for Modification of Chemical Fibers and Polymer Materials, College of Materials Science and Engineering, Donghua University, Shanghai, 201620 China

**Keywords:** Prostate cancer, Inflammatory markers, Prognosis, Meta-analysis

## Abstract

**Background:**

Pretreatment inflammatory factors, including neutrophil, lymphocyte, platelet and monocyte counts as well as the ratios between them such as neutrophil–lymphocyte ratio (NLR), platelet–lymphocyte ratio (PLR) and lymphocyte–monocyte ratio (LMR) have been suggested as potential prognostic predictors for patients with prostate cancer (PCa). However, the prognostic effects remain controversial. Therefore, the goal of this study was evaluate the prognostic values of these markers for PCa patients using a meta-analysis.

**Methods:**

Potentially relevant publications in PubMed and Cochrane Library were searched. Pooled hazard ratio (HR) with 95% confidence interval (CI) for overall survival (OS), cancer-specific survival (CSS), progression-free survival (PFS), recurrence free survival (RFS) and distant metastases-free survival (DMFS) were determined using a fixed or random effects model by STATA 13.0 software.

**Results:**

Thirty-two studies involving 21,949 participants were included. Our pooled results demonstrated that a high pretreatment NLR (HR = 1.55, 95% CI 1.37–1.76), PLR (HR = 1.72; 95% CI 1.36–2.18), neutrophil (HR = 1.10; 95% CI 1.03–1.18 and monocyte counts (HR = 2.25; 95% CI 1.67–3.05) predicted inferior OS, while elevated pretreatment LMR (HR = 2.27; 95% CI 1.76–2.94) was correlated with favorable OS. Furthermore, the higher NLR (HR = 1.62; 95% CI 1.29–2.04) and monocyte counts (HR = 1.75; 95% CI 1.36–2.25), but lower LMR predicted worse PFS (HR = 2.18; 95% CI 1.58–3.02); poor RFS was only associated with NLR (HR = 1.12; 95% CI 1.04–1.20). The subgroup analysis showed that the higher NLR may be a predictive factor for OS only in patients with mCRPC and undergoing chemotherapy; while the higher PLR was only significantly associated with OS in localized PCa regardless of treatment.

**Conclusion:**

This meta-analysis reveals that pretreatment NLR, PLR, LMR, neutrophil, and monocyte counts may be effective predictive biomarkers for prognosis in patients with PCa.

## Background

Prostate cancer (PCa) represents a considerable health concern for the male population, with an estimated 161,360 new cases diagnosed and 26,730 death in 2017 in the United States [1]. Radical prostatectomy, androgen deprivation therapy, radiotherapy and chemotherapy have been recommended for treatment of patients with PCa, however, the overall survival (OS) rate remains unsatisfactory (5-year: 77.52%; 10-year: 62.57%) due to tumor local recurrence, progression or distal metastasis [2]. Therefore, how to stratify the high risk patients who will have poor prognosis because of non-response to the treatment strategies and prone to recurrence, progression or metastasis has become a hot issue urgently needed to be solved.

Conventionally, prognosis of patients with PCa can be predicted by pathologic tumor, node, metastasis (TNM) staging, prostate-specific antigen (PSA) level [3], PSA doubling time [3] and Gleason score [4]. Nevertheless, some clinical studies reported that patients with the same stage may have different prognoses [5] and prognosis seemed to be similar among patients with different PSA levels [6], indicating the insufficient accuracy of these factors for prognosis prediction. Thus, supplementary pretreatment biomarkers should be developed to assist clinicians to evaluate prognosis and further guide decision-making concerning therapeutic strategies.

Recently, accumulating evidences suggest inflammation may play important roles in the development and progression of PCa [7, 8]. Neutrophil, lymphocyte, platelet (PLT) and monocyte counts as well as the ratios between them such as neutrophil–lymphocyte ratio (NLR), platelet–lymphocyte ratio (PLR) and lymphocyte–monocyte ratio (LMR) are the common, indicators for the inflammatory status in patients with cancer [9–11]. Also, the collection of these indicators in blood is simple, noninvasive, and easily accessible. Hereby, circulating inflammatory factors may represent potential prognostic biomarkers for PCa in clinic. This hypothesis has been demonstrated by several studies: elevated NLR [12], PLR [13] and lower LMR [14] were found to be significantly associated with worse OS. However, a handful of studies demonstrated NLR and PLR may be ineffective markers for predicting PCa prognosis [15–17] or contrast conclusions were achieved (that is, higher NLR [18, 19] may be protective factors for prognosis). These controversial conclusions may be attributed to the different study design and small sample size. Thus, it is necessary to further evaluate the prognostic significance of the above inflammatory biomarkers for patients with PCa by performing a meta-analysis that can integrate all related articles. Compared with the recently published meta-analysis that included the articles up to July 2015 [20], February 2016 [21] for NLR and March 2017 for PLR [22], our study may collect more literatures by searching the relevant databases until August 2018, which may lead to more survival index predicted and a more convinced conclusion achieved. In addition, to our knowledge, there was no a pooled study to assess the prognostic significance of the neutrophil, lymphocyte, platelet and monocyte counts and LMR in PCa until now.

Given the newly emerging evidence, the goal of this study was to further conduct a systematic review and meta-analysis to reveal the prognostic performance (OS; PFS, progression-free survival; CSS, cancer-specific survival; DMFS, distant metastases-free survival; RFS, recurrence-free survival) of all well-known systemic inflammatory parameters for all patients with PCa and subgroups with different treatments. Pretreatment identification of the level or ratio of these biomarkers may be beneficial for the stratification of prognosis and schedule of medical treatments.

## Materials and methods

### Literature search strategy

This meta-analysis was performed in accordance to the Guidelines of the Preferred Reporting Items for Systematic Review and Meta-Analysis (PRISMA) [23, 24] (no review protocol existed previously).

A systematic literature search was carried out by using PubMed and Cochrane Library databases to assess the relationship between pretreatment systemic inflammatory biomarkers (NLR, PLR, LMR, platelet, lymphocyte, monocyte and neutrophil) and the prognosis of patients with PCa. The current search was updated to August 2018 using the combinations of the following keywords: (1) ‘NLR’ (or “neutrophil to lymphocyte ratio” or “neutrophil lymphocyte ratio” or “neutrophil-to-lymphocyte”); OR (2) ‘PLR’ (or “platelet to lymphocyte ratio” or “platelet lymphocyte ratio” or “platelet-to-lymphocyte”; OR (3) ‘LMR’ (or “lymphocyte monocyte ratio” or “lymphocyte-monocyte ratio” or “lymphocyte to monocyte ratio”); OR (4) ‘platelet’; OR (5) ‘lymphocyte’; OR (6) ‘monocyte’; OR (7) ‘neutrophil’; AND ‘prostatic cancer’ (or “prostate carcinoma” or “prostatic carcinoma”). Furthermore, the reference lists of all identified publications as well as pertinent reviews and meta-analyses were also manually inspected to further screen potentially eligible articles.

### Selection criteria

Two investigators (HP and XGL) independently screened the candidate publications from the databases. Few disagreements were resolved by discussion and consensus. Literatures were considered eligible if they met the following inclusion criteria: (1) PCa was confirmed by pathological examination; (2) pretreatment (such as chemoradiotherapy, surgery) biomarkers (NLR, PLR, LMR, PLT, lymphocyte, monocyte and neutrophil) were measured by serum-based method and calculated by standard formula with same unit; (3) prognostic related outcomes [such as OS (all-cause mortality), PFS, CSS, DMFS and RFS] were investigated; (4) hazard ratio (HR) with a 95% confidence interval (CI) could be directly obtained or indirectly calculated. Studies were excluded if they were: (1) duplicated literatures; (2) abstracts, letters, reviews, editorials, case reports, comments or non-clinical studies; (3) lack of insufficient data to estimate HR and 95% CI; and (4) literature written in language other than English.

### Data extraction

Two investigators (HP and XGL) independently extracted the following data: name of first author, publication year, country, sampling time, sample size, study design, patient characteristics (including gender, age, patient status, duration of follow-up), treatment details, cut-off value, HR with 95% CI for survival and associated statistical methods. HR and 95% CI were extracted preferentially from multivariable analyses where available. If not, univariate analysis results were collected. Disagreements were resolved through discussion to reach consensus.

The quality of the included studies was assessed using the Newcastle–Ottawa Scale (NOS) [25] that consists of three domains: patients selection (0–4 points), comparability (0–2 points), and outcome assessment (0–3 points). Studies with the scores ≥ 7 were considered to be of high-quality.

### Statistical analysis

Statistical heterogeneity among the studies was tested by using Cochrane’s Q (Chi squared) and *I*^2^ statistic [26]. A random-effects model was chosen to calculate the pooled HR and 95% CI for heterogeneous studies (Q test P value < 0.10 and *I*^2^ > 50%); otherwise, the fixed-effects model was used. Publication bias was assessed with Egger’s linear regression test with funnel plots [27]. The influence of publication bias on the overall effect was evaluated by the ‘‘trim and fill’’ method [28]. Sensitivity analysis was performed by recalculating the pooled HRs after omitting one study in each turn from the meta-analysis consecutively (leave-one-out procedure). In addition, a subgroup analysis was also performed according to stratification of ethnicity, sample size, study design, patient status, follow-up time, cut-off, statistical methods and therapy. All statistical analyses were conducted using the STATA software (version 13.0; STATA Corporation, College Station, TX, USA). *P* < 0.05 was set as the statistical significance level.

## Results

### Study characteristics

**Fig. 1:**
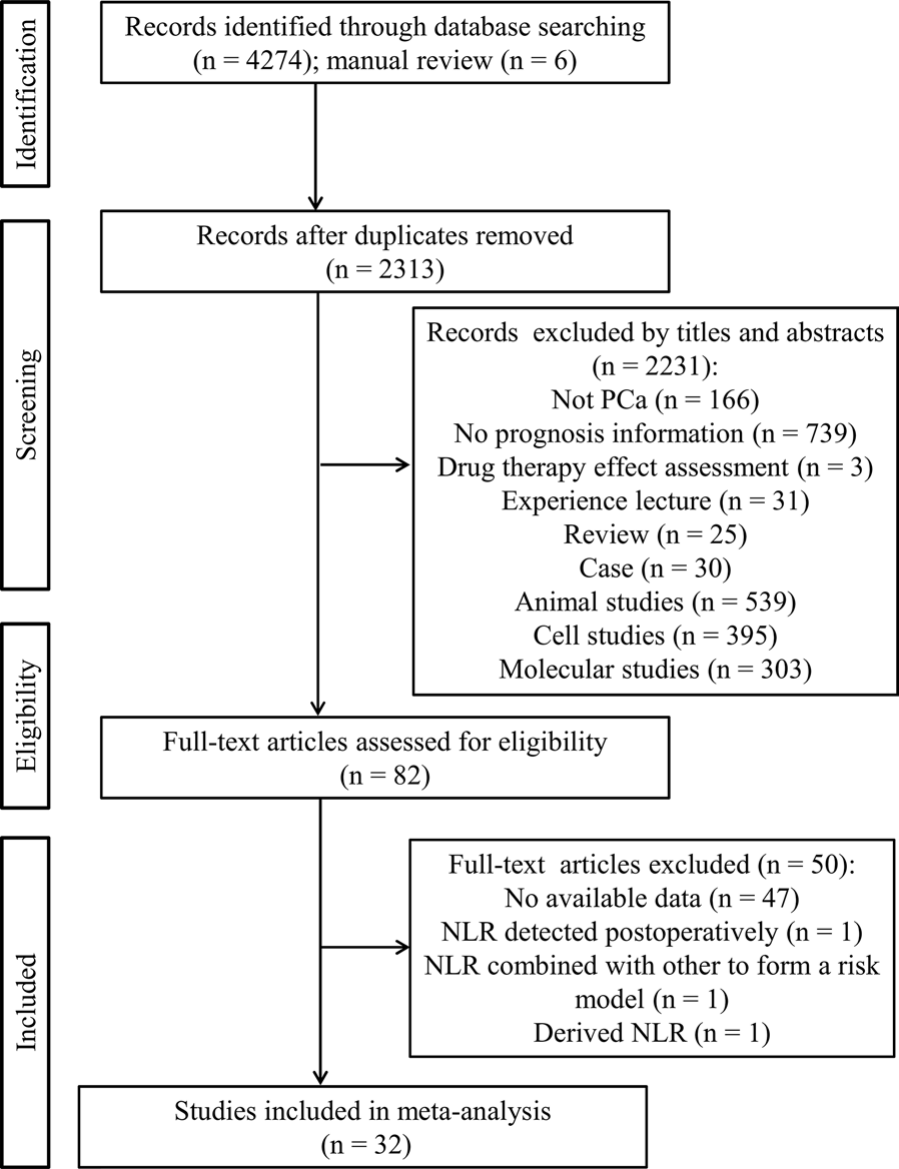
Flow diagram of study identification

A flow chart of the literature search is presented in Fig. 1. The initial search yielded 4280 studies based on the search strategies, in which 1967 were excluded as they were duplicates. After title and abstract screening, 2231 studies were excluded because the following reasons: not PCa (n = 166), no prognosis information (n = 739), drug therapy effect assessment (n = 3), experience lecture (n = 31), review (n = 25), case (n = 30), animal studies (n = 539), cell studies (n = 395) and molecular studies (n = 303). Eighty-two full-text articles were then downloaded to assess their eligibility, in which 54 were further excluded because non-effective data could be collected (n = 47), NLR was detected postoperatively (n = 1) and NLR was combined with other to form a risk model (n = 1) and derived NLR (dNLR) was demined (n = 1). Ultimately, 32 studies (n = 21,949 participants) were included for subsequent meta-analysis [12–16, 18, 19, 29–52], of which 28 studies including 21,452 participants were included for NLR [12–16, 18, 19, 29–38, 40–43, 45–47, 50–52], 6 studies including 2994 participants for PLR [13, 17, 30, 37, 39, 44], 4 studies including 2594 participants for PLT [13, 30, 39, 48], 4 studies including 5133 participants for lymphocyte counts [30, 36, 39, 41], 3 studies including 4843 participants for neutrophil counts [30, 36, 41], 2 studies involving 504 participants for monocyte counts [14, 49] and 1 study involving 214 participants for LMR [14]. The study of Shigeta et al. [14] had two datasets (training and validation) and both of them were included for assessment of the prognostic value of NLR, LMR and monocyte for PCa. The other characteristics of the included studies are shown in Table 1. NOS scores of all of the selected articles were ≥ 7, suggesting high quality (Table 2).

**Table 1:** Characteristics of all the studies included in the meta-analysis

Study	Year	Country	Time	No.	Age (years)	Index and cut-off	Median follow-up (month)	Outcome
Yasui	2018	Japan	2011–2016	90	73.7 ± 0.9	NLR: 3.76	Unclear	OS
Fan	2018	China	2013–2017	104	72 (65.3–77.0)	NLR: 3	20.2	OS, PFS
Conteduca	2018	Italy	2011–2016	551	75 (42–91)	NLR: 3	18.4	OS, PFS
Vidal	2018	USA	1991–2015	1826	61.8 ± 5.9	NLR; lymphocyte; PLT; neutrophil; PLR	68	OS, RFS
Sun	2018	China	2011–2016	171	Unclear	NLR: 2.31; PLR:134	24	OS, DFS
Uemura	2017	Japan	2014–2016	47	71.0 ± 7.0	NLR: 3.83	Unclear	OS
Mehra	2017	UK	Unclear	75	Unclear	NLR: 2.6	25.6	OS, PFS
Boegemann	2017	USA	2009–2015	96	70 (63.0–76.3)	NLR: 5	20	OS, PFS
Pei	2017	China	2013–2017	111	71 (43–86)	NLR: 3.3	16	OS, PFS
Buttigliero	2017	Italy	Unclear	110	Unclear	NLR: 3	31.7	OS, PFS
Wang	2017	China	2010–2014	290	71 (65–77)	Monocyte:0.425	37.0	OS, PFS
Jang	2016	Korea	2000–2010	2067	66 (61–70)	NLR:1.76; lymphocyte, neutrophil	78	OS, RFS, CSS
Lolli	2016	Italy	2011–2015	230	74 (45–90)	NLR: 3; PLR:210	29	OS
Shigeta	2016	Japan	2007–2015	108 106	71 (57–88) 73 (52–95)	NLR: 3.8; LMR: 3.86; monocyte: 0.4	Unclear	OS, PFS
Lee	2016	Korea	Unclear	1367	65.6 (45–82)	NLR: 2.5	57	RFS
Wang	2016	China	2010–2014	290	75 (67–79)	Lymphocyte; PLT:190.5; PLR:117.58	37	OS, PFS, CSS
Langsenlehner	2015	Austria	1999–2007	415	66.9 ± 7.2	NLR: 5	87	OS, PFS, DMFS
Langsenlehner	2015	Austria	1999–2007	374	68 ± 7.1	NLR; PLT; PLR:190	87	OS, CSS, DMFS
Zhang	2015	China	2006–2009	237	Unclear	NLR: 2.36	46.6	RFS
Bahig	2015	Canada	2001–2014	950	Unclear	NLR; lymphocyte; neutrophil	44	OS, RFS
Lorente	2015	UK	Unclear	755	67 (62–73)	NLR: 3	12.8	OS, PFS
Yao	2015	Japan	2008–2014	57	74 (55–91)	NLR: 3.5	30	OS, PFS
Li	2015	China	2009–2012	103	Unclear	PLR:150	36	OS
McLachlan	2015	Austria	2005–2012	42	Unclear	NLR:5	23.1	OS
Sharma	2015	USA	1990–2007	8350	Unclear	NLR:5	116.4	OS, PFS, RFS, CSS
Templeton	2014	Canada	2001–2011	357	71.0 (44.0–90.0)	NLR: 3	Unclear	OS
Sonpavde	2014	UK	2008–2010	848	Unclear	NLR:2.5	Unclear	OS, RFS
Nuhn	2014	USA	1998–2010	238	68.3 (44.6–84.5)	NLR: 3	15.0	OS
Poyet	2013	Switzerland	2008–2013	399	64.0 (41.0–78.0)	NLR: 2.67	23.0	RFS
Linton	2013	Austria	2007–2009	184	Unclear	NLR: 5	Unclear	OS
Shafique	2012	UK	2000–2007	897	Unclear	NLR: 5	30	OS
Yamada	2011	Japan	1998–2006	104	74.2 ± 7.4	PLT	43	CSS

*OS* overall survival, *CSS* cancer-specific survival, *PFS* progression-free survival, *DMFS* distant metastases-free survival, *RFS* recurrence-free survival, *PLR* platelet to lymphocyte ratio, *NLR* neutrophil to lymphocyte ratios, *LMR* lymphocyte to monocyte ratio, *PLT* platelet

**Table 2:** Quality assessment scale for 32 studies

Study	Selection				Comparability		Outcome			Total scores
	Representativeness of the exposed cohort	Selection of the non-exposed cohort	Assessment of exposure	Outcome not present at start of study	Comparability of cohorts on the basis		Assessment of outcome	Follow-up long enough for outcome	Adequacy of follow-up	
					Design	Analysis				
Yasui	+	+	+		+		+	+	+	7
Fan	+	+	+	+	+	+	+	+	+	9
Conteduca	+	+	+	+	+	+	+	+	+	9
Vidal	+	+	+		+		+	+	+	7
Sun	+	+	+		+	+	+	+	+	8
Uemura	+	+	+		+		+		+	7
Mehra	+	+	+	+	+	+	+	+	+	9
Boegemann	+	+	+		+	+	+	+	+	8
Pei	+	+	+		+	+	+	+	+	8
Buttigliero	+	+	+		+		+	+	+	7
Wang	+	+	+	+	+		+	+	+	7
Jang	+	+	+		+	+	+	+	+	8
Lolli	+	+	+		+	+	+	+	+	8
Shigeta	+	+	+		+		+	+	+	7
Lee	+	+	+		+	+	+	+	+	8
Wang	+	+	+		+	+	+	+	+	8
Langsenlehner	+	+	+		+	+	+	+	+	8
Langsenlehner	+	+	+		+	+	+	+	+	8
Zhang	+	+	+		+		+	+	+	7
Bahig	+	+	+		+	+	+	+	+	8
Lorente	+	+	+		+	+	+	+	+	9
Yao	+	+	+		+	+	+	+	+	8
Li	+	+	+		+		+	+	+	7
McLachlan	+	+	+		+		+	+	+	7
Sharma	+	+	+		+		+	+	+	7
Templeton	+	+	+		+		+	+	+	7
Sonpavde	+	+	+		+	+	+		+	7
Nuhn	+	+	+		+	+	+	+	+	8
Poyet	+	+	+		+		+	+	+	7
Linton	+	+	+	+	+	+	+	+	+	9
Shafique	+	+	+	+	+	+	+	+	+	9
Yamada	+	+	+		+	+	+	+	+	8

A positive result on any one of them was counted as one point

### Association between NLR and PCa survival

**Fig. 2:**
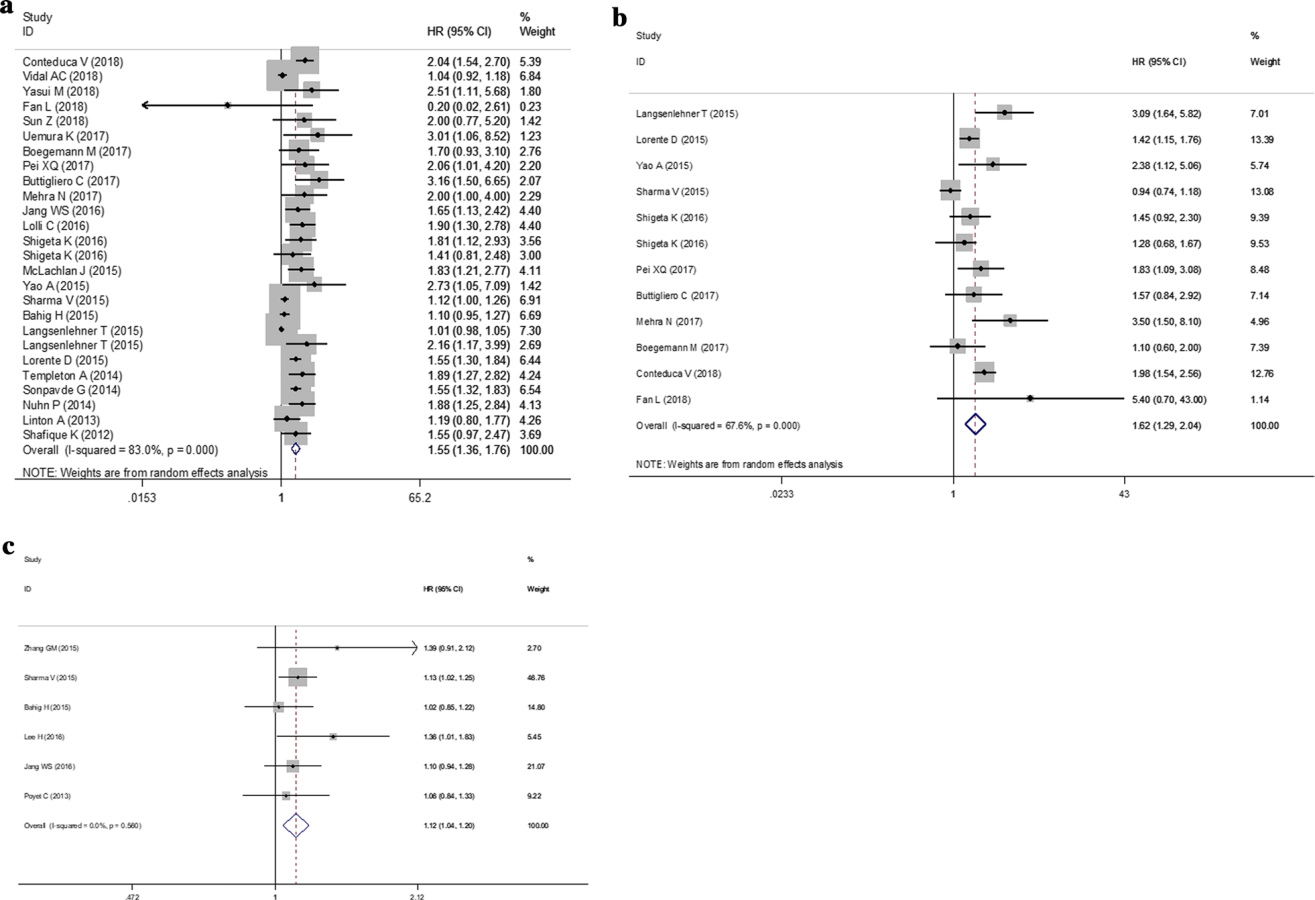
Forest plots of the significant correlations of neutrophil to lymphocyte ratio with survival. **a** Overall survival; **b** progression-free survival; **c** recurrence-free survival. Squares are hazard ratio (HR); horizontal lines are 95% confidence intervals (CI); blue diamond indicates the pooled HR estimate with its 95% CI

Twenty-five studies with 26 datasets evaluated the association between pretreatment NLR and OS in PCa patients. A significant heterogeneity was present among studies (*I*^2^ = 83.4%, *P* < 0.001) and thus a random-effects model was chosen to pool the study results. The synthesized estimates analysis showed that patients with elevated NLR had significantly shorter OS (HR = 1.55, 95% CI 1.37–1.76, *P* < 0.001) (Fig. 2a; Table 3).

**Table 3:** Overview of pooled results of the prognostic value of hematologic parameters

	Outcome	No.	HR	95% CI	*P*	I-squared (%)	*P*_H_
NLR	OS	26	1.55	1.37–1.76	0.000	83.0	0.000
	CSS	4	1.14	0.89–1.45	0.297	61.5	0.050
	PFS	12	1.62	1.29–2.04	0.000	67.6	0.000
	DMFS	2	1.81	0.55–6.01	0.330	92.3	0.000
	RFS	6	1.12	1.04–1.20	0.002	0.0	0.560
PLR	OS	6	1.72	1.36–2.18	0.000	0.0	0.575
	CSS	3	1.56	0.82–2.97	1.000	78.7	0.009
PLT	OS	3	1.00	0.98–1.02	0.910	59.3	0.086
	CSS	4	1.00	0.99–1.01	0.850	15.4	0.315
LMR	OS	2	2.27	1.76–2.94	0.000	27.8	0.239
	PFS	2	2.18	1.58–3.02	0.000	0.0	0.460
Lymphocyte	OS	4	0.96	0.83–1.10	0.710	0.0	0.529
	CSS	3	0.85	0.66–1.09	0.190	0.0	0.523
	RFS	2	1.06	0.96–1.16	0.260	0.0	0.824
Neutrophil	OS	3	1.10	1.03–1.18	0.006	0.0	0.604
	CSS	2	1.12	0.99–1.25	0.065	0.0	0.771
	RFS	2	1.03	0.98–1.09	0.188	0.0	0.421
Monocyte	OS	3	2.25	1.67–3.05	0.000	1.54	0.463
	PFS	3	1.75	1.36–2.25	0.000	33.2	0.224

OS, overall survival; CSS, cancer-specific survival; PFS, progression-free survival; DMFS, distant metastases-free survival; RFS, recurrence-free survival; PLR, platelet to lymphocyte ratio; NLR, neutrophil to lymphocyte ratio; LMR, lymphocyte to monocyte ratio; PLT, platelet; HR, hazard ratio; CI, confidence intervals; *P*_H_, heterogeneity of *P*-value

There were 4 studies examined the relationship of pretreatment NLR with CSS in PCa patients. A significant heterogeneity was detected among studies (*I*^2^ = 61.5%, *P* = 0.05) and thus a random-effects model was applied. The estimated pooled HR (95% CI) of 1.14 (95% CI 0.89–1.45, *P* = 0.297) in 3 studies indicated that patients with an elevated NLR were not associated with shorter CSS after treatment (Table 3).

Eleven studies with 12 datasets analyzed the pretreatment NLR for predicting the PFS of PCa after treatment. A random-effects model was adopted to pool the study results because the *I*^2^ = 67.6% and *P* < 0.001. The pooled estimates analysis predicted PFS was significantly lower in PCa patients with an elevated NLR (random: HR = 1.62; 95% CI 1.29–2.04, *P* < 0.001) (Fig. 2b; Table 3).

There were 2 studies to investigate the relationship between pretreatment NLR and DMFS in PCa patients. The Cochran’s Q-test resulted in a *P* value < 0.001 and the corresponding *I*^2^ was 92.3%, indicating that there was significant heterogeneity among studies. A random-effects model analysis demonstrated no statistically significant difference in DMFS of patients with higher or lower NLR (HR = 1.81; 95% CI 0.55–6.01, *P* = 0.330; Table 3).

Six studies analyzed the relationship between pretreatment NLR and RFS in PCa patients. There was no evidence of heterogeneity among studies because the Cochran’s Q-test P-value was 0.560 and the corresponding *I*^2^ was 0%. A fixed-effects model analysis demonstrated the higher level of NLR indicated unfavorable RFS (HR = 1.12; 95% CI 1.04–1.20, *P* = 0.002) (Fig. 2c; Table 3).

### Association between PLR and PCa survival

**Fig. 3:**
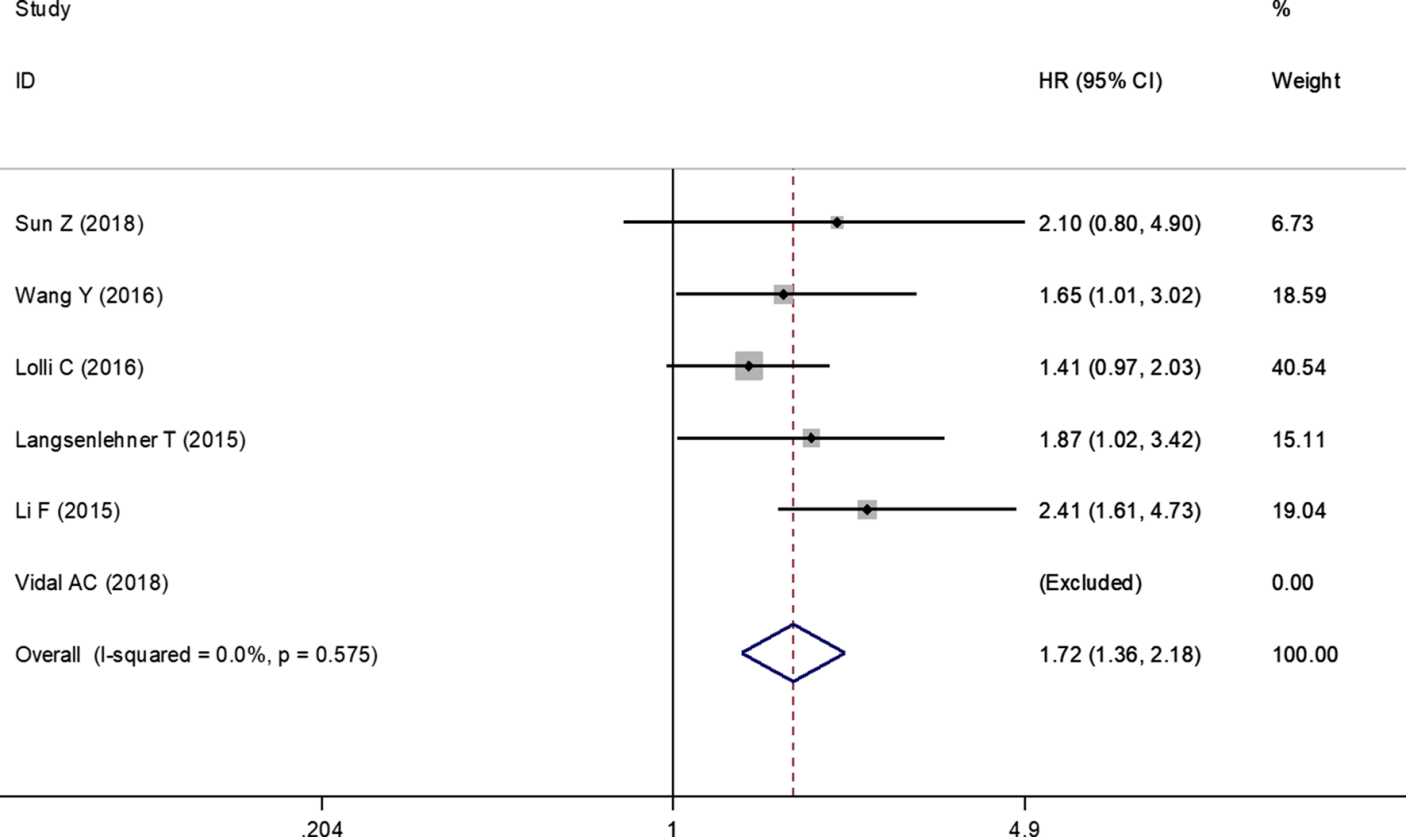
Forest plots of the significant correlation of platelet to lymphocyte ratio with overall survival. Squares are hazard ratio (HR); horizontal lines are 95% confidence intervals (CI); blue diamond indicates the pooled HR estimate with its 95% CI

Six studies evaluated the association between pretreatment PLR and OS in PCa patients. No significant heterogeneity was present among studies (*I*^2^ = 0%, *P* = 0.575) and thus a fixed-effects model was chosen. The pooled analysis showed that higher level of PLR was associated with shorter OS (HR = 1.72; 95% CI 1.36–2.18, *P* < 0.001) (Fig. 3; Table 3).

There were 3 studies examined the relationship of pretreatment PLR with CSS in PCa patients. A significant heterogeneity was detected among studies (*I*^2^ = 78.7%, *P* = 0.009) and thus a random-effects model was applied. The estimated pooled HR (95% CI) of 1.56 (95% CI 0.82–2.97, *P* = 0.174) in 3 studies indicated that patients with an elevated PLR were not associated with shorter CSS after treatment (Table 3).

### Association between LMR and PCa survival

**Fig. 4:**
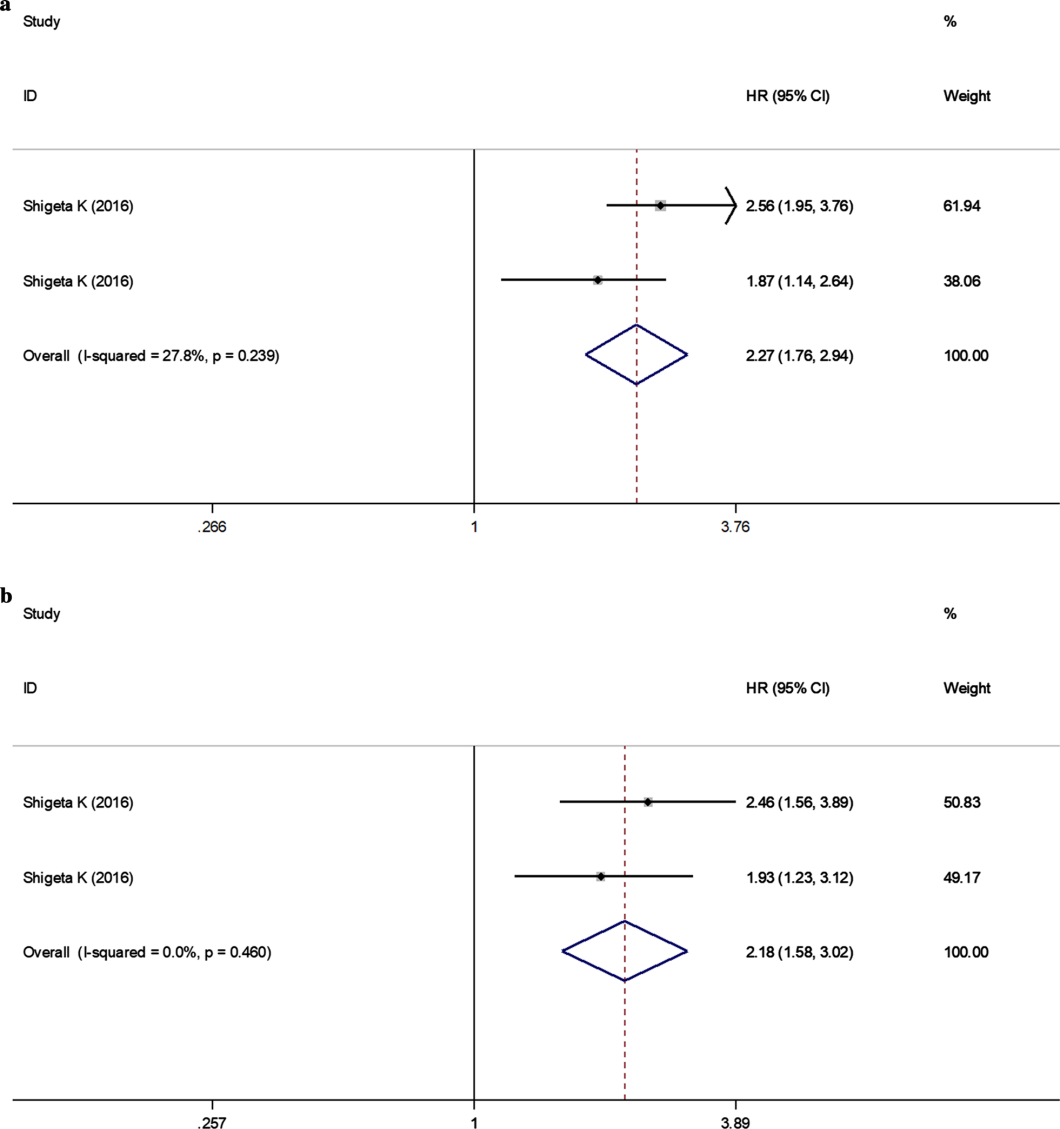
Forest plots of the significant correlation of lymphocyte to monocyte ratio with survival. **a** Overall survival; **b** progression-free survival. Squares are hazard ratio (HR); horizontal lines are 95% confidence intervals (CI); blue diamond indicates the pooled HR estimate with its 95% CI

One study with 2 datasets analyzed the pretreatment LMR for predicting the OS of PCa after treatment. No significant heterogeneity was present among studies (*I*^2^ = 27.8%, *P *= 0.239) and thus a fixed-effects model was chosen. The pooled analysis showed that higher level of LMR was associated with more favorable OS (HR = 2.27; 95% CI 1.76–2.94, *P* < 0.001) (Fig. 4a; Table 3).

One study with 2 datasets analyzed the pretreatment LMR for predicting the PFS of PCa after treatment. No significant heterogeneity was present among studies (*I*^2^ = 27.8%, *P* = 0.460) and thus a fixed-effects model was chosen. The pooled analysis showed that higher level of LMR was associated with longer PFS (HR = 2.18; 95% CI 1.58–3.02, *P* < 0.001) (Fig. 4b; Table 3).

### Association between PLT and PCa survival

Three studies evaluated the association between pretreatment PLT and OS in PCa patients. There was evidence of heterogeneity among studies (*I*^2^ = 56.1%, P = 0.086) and thus a random-effects model was chosen. The pooled analysis showed that pretreatment PLT was not associated with OS (HR = 1.00; 95% CI 0.98–1.02, *P* = 0.910; Table 3).

Four studies evaluated the association between pretreatment PLT and CSS in PCa patients. There was no evidence of heterogeneity among studies (*I*^2^ = 15.4%, *P* = 0.315) and thus a fixed-effects model was chosen. The pooled analysis showed that pretreatment PLT was not associated with CSS (HR = 1.00; 95% CI 0.99–1.01, *P* = 0.850; Table 3).

### Association between lymphocyte counts and PCa survival

Four articles studied the association between pretreatment lymphocyte counts and OS in PCa patients. There was no evidence of heterogeneity among studies (*I*^2^ = 0%, *P* = 0.710) and thus a fixed-effects model was chosen. The pooled analysis showed that pretreatment lymphocyte counts could not predict the OS (HR = 0.96; 95% CI 0.83–1.10, *P* = 0.529; Table 3).

Three articles provided CSS as the primary outcome. There was no evidence of heterogeneity among studies (*I*^2^ = 0%, *P* = 0.523) and thus a fixed-effects model was chosen. The pooled analysis showed that the higher lymphocyte counts also could not predict the CSS (HR = 0.85; 95% CI 0.66–1.09, *P* = 0.190; Table 3).

Two articles studied the association between pretreatment lymphocyte counts and RFS in PCa patients. There was no evidence of heterogeneity among studies (*I*^2^ = 0%, *P *= 0.824) and thus a fixed-effects model was chosen. The pooled analysis showed that pretreatment lymphocyte counts were similarly not associated with RFS (HR = 1.06; 95% CI 0.96–1.16, *P* = 0.260; Table 3).

### Association between neutrophil counts and PCa survival

**Fig. 5:**
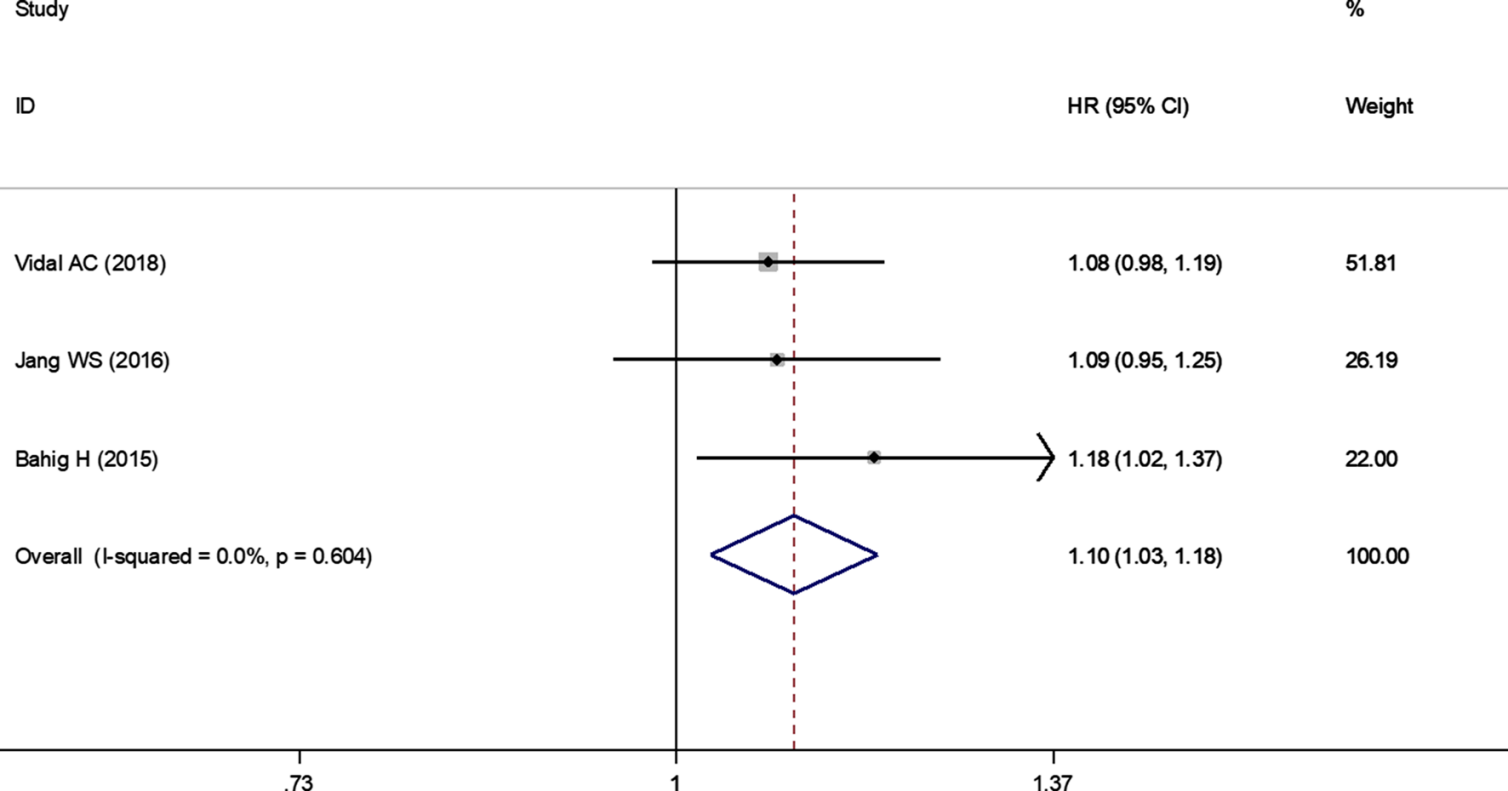
Forest plots of the significant correlation of neutrophil counts with overall survival. Squares are hazard ratio (HR); horizontal lines are 95% confidence intervals (CI); blue diamond indicates the pooled HR estimate with its 95% CI

Three articles studied the association between pretreatment neutrophil counts and OS in PCa patients. There was no evidence of heterogeneity among studies (*I*^2^ = 0%, *P* = 0.604) and thus a fixed-effects model was chosen. The pooled analysis showed that patients with higher pretreatment neutrophil counts were expected to have shorter OS (HR = 1.10; 95% CI 1.03–1.18, *P* = 0.006) (Fig. 5; Table 3).

Two articles provided sufficient data on CSS outcome for the pooled estimate of neutrophil counts in PCa patients. There was no evidence of heterogeneity among studies (*I*^2^ = 0%, *P *= 0.771) and thus a fixed-effects model was chosen. The pooled analysis showed that the higher neutrophil counts did not predict poorer CSS (HR = 1.12; 95% CI 0.99–1.25, *P* = 0.065) (Table 3).

Two articles studied the association between pretreatment neutrophil counts and RFS in PCa patients. There was no evidence of heterogeneity among studies (*I*^2^ = 0%, *P* = 0.942) and thus a fixed-effects model was chosen. The pooled analysis showed no significant difference in neutrophil counts observed between patients with higher and lower pretreatment neutrophil counts (HR = 1.03; 95% CI 0.98–1.09, *P* = 0.188; Table 3).

### Association between monocyte counts and PCa survival

**Fig. 6:**
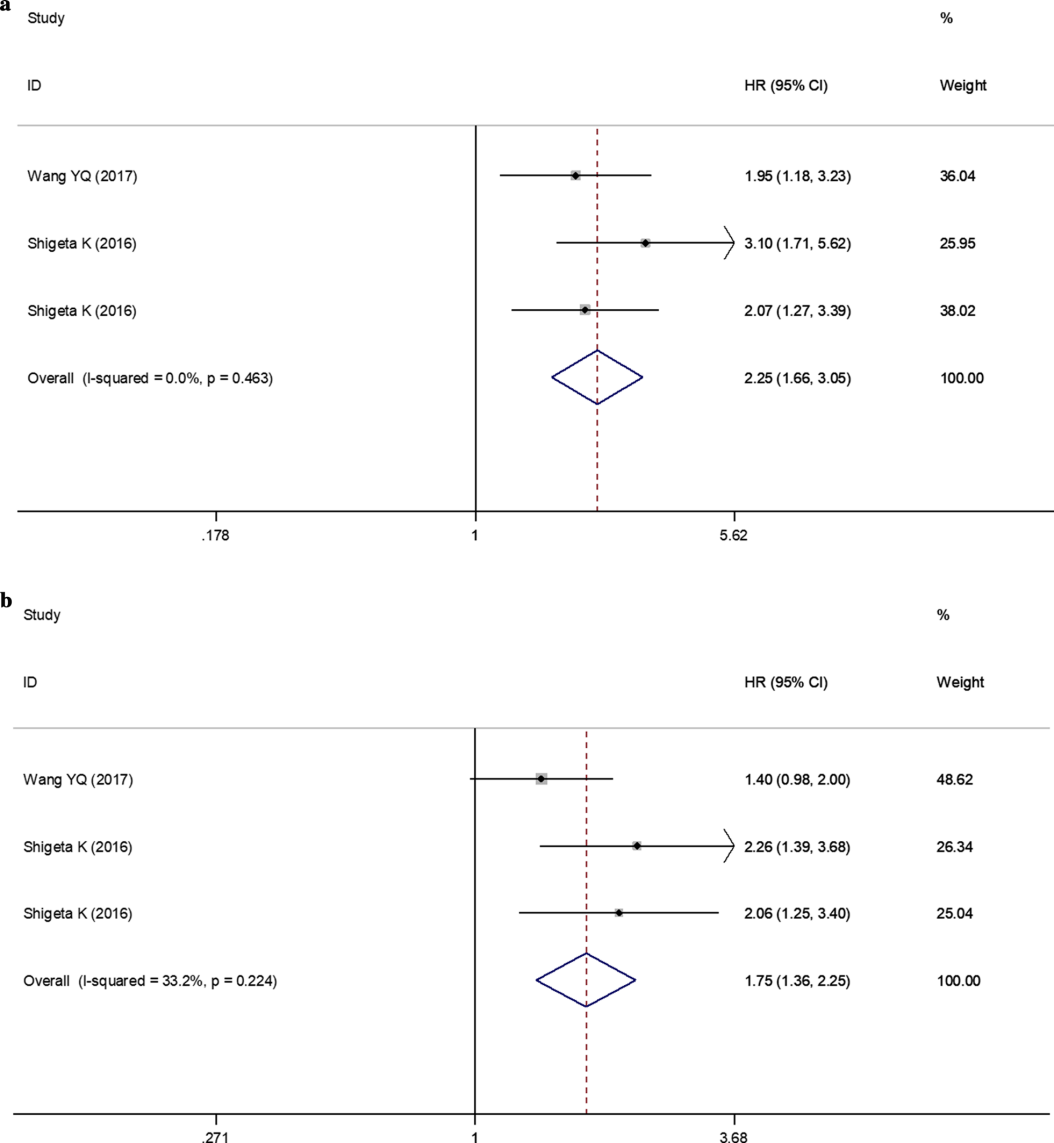
Forest plots of the significant correlation of monocyte counts with survival. **a** Overall survival; **b** progression-free survival. Squares are hazard ratio (HR); horizontal lines are 95% confidence intervals (CI); blue diamond indicates the pooled HR estimate with its 95% CI

Two articles with three datasets studied the association between pretreatment monocyte counts and OS in PCa patients. There was no evidence of heterogeneity among studies (*I*^2^ = 0%, *P *= 0.463) and thus a fixed-effects model was chosen. The pooled analysis showed that patients with higher pretreatment monocyte counts were expected to have shorter OS (HR = 2.25; 95% CI 1.67–3.05, *P* < 0.001) (Fig. 6a; Table 3).

Two articles with three datasets provided sufficient data on PFS outcome for the pooled estimate of monocyte counts in PCa patients. There was no evidence of heterogeneity among studies (*I*^2^ = 33.2%, *P* = 0.224) and thus a fixed-effects model was chosen. The pooled analysis showed that the higher monocyte counts predicted poorer PFS (HR = 1.75; 95% CI 1.36–2.25, *P* < 0.001) (Fig. 6b; Table 3).

### Publication bias

**Fig. 7:**
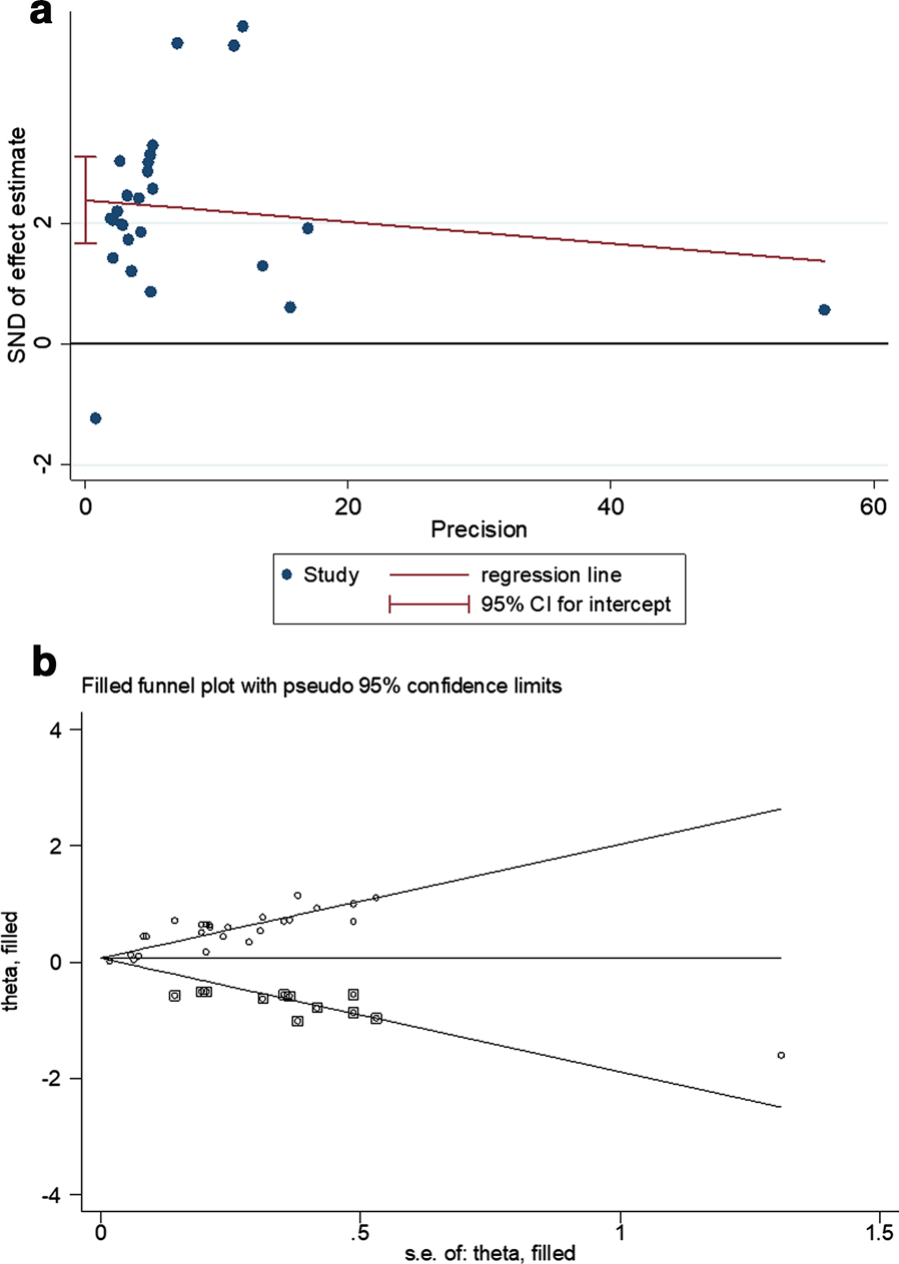
Funnel plot for the assessment of potential publication bias. **a** Egger’s funnel plot for overall survival of NLR; **b** Trim-and-fill funnel plot for overall survival of NLR. *SND* standard normal deviation, *s.e.* standard error, *CI* confidence intervals

The above analysis showed that NLR, PLR, neutrophil and monocyte counts were hematologic parameters significantly associated with prognosis (NLR: OS, PFS and RFS; PLR: OS; neutrophil counts: OS; monocyte counts: OS and PFS). Therefore, funnel plots of the pooled analysis of these 4 parameters were constructed to examine whether there was a publication bias. The results revealed that the publication bias was present in NLR for OS (*P* < 0.001) (Fig. 7a), but not in NLR for PFS (*P* = 0.189) and RFS (*P* = 0.398); PLR for OS (*P* = 0.218); neutrophil counts for OS (*P* = 0.454); monocyte counts for OS (*P* = 0.173) and PFS (*P* = 0.137). Subsequently, a trim-and-fill method was performed to explore the influence of publication bias on the effect estimate. The filled meta-analysis still indicated there was still a significant association between pretreatment NLR and OS (HR = 1.21; 95% CI 1.07–1.37, *P* = 0.007) (Fig. 7b). Only one study was included for LMR and thus publication bias was not present.

### Sensitivity analyses

**Fig. 8:**
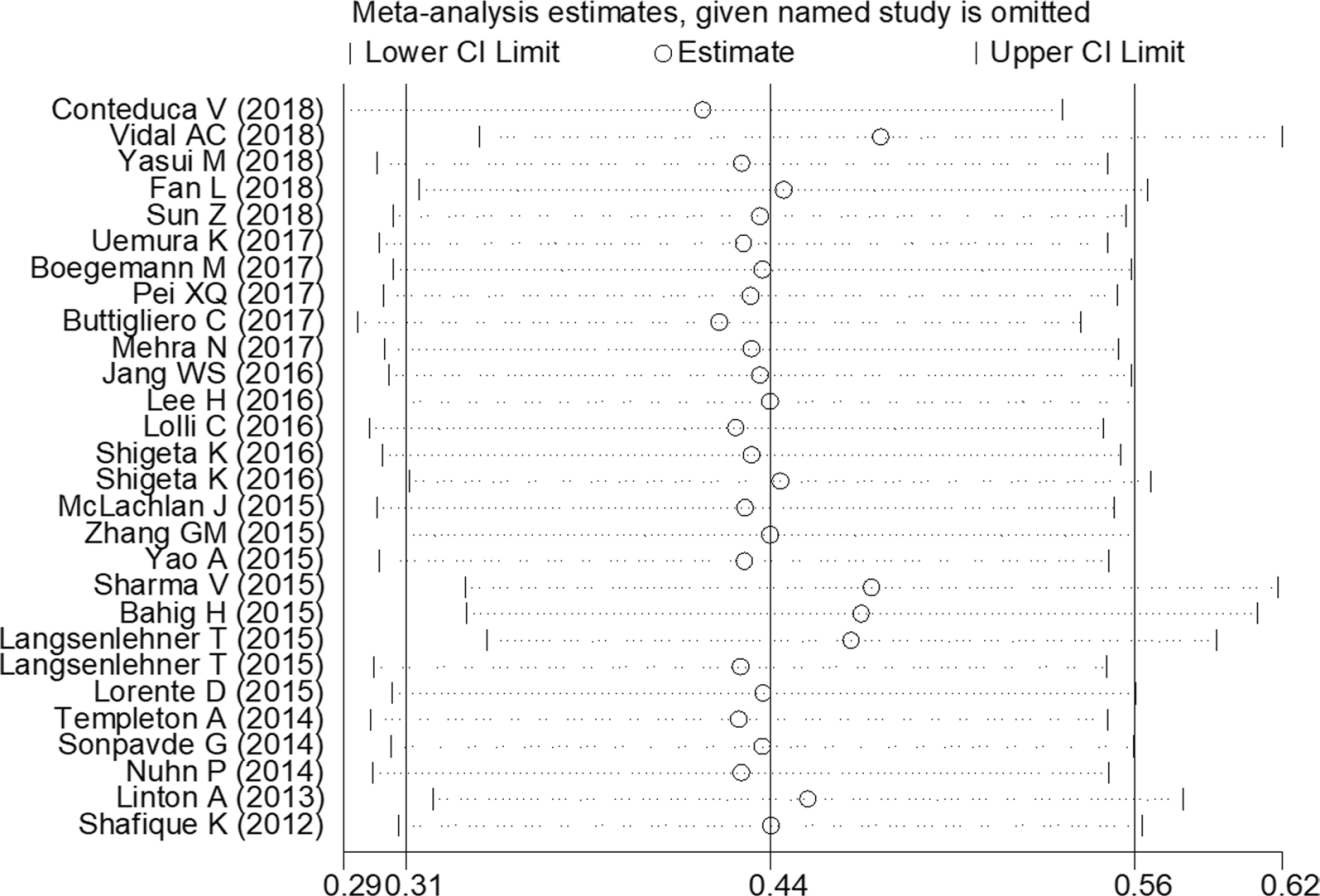
Sensitivity analysis. The horizontal axis was ln(HR). The two ends of every broken line represent the 95% CI

Sensitivity analysis was performed by assessing the potential impact of individual dataset on the pooled data. The results showed that pooled HR was not significantly altered when each single study was deleted every time (Fig. 8).

### Subgroup analysis

The subgroup analyses according to ethnicity, sample size, study design, patient status, follow-up time, cut-off, statistical methods and therapy were only performed for NLR and PLR due to limited literatures. The results showed that elevated NLR predicted poor prognosis in almost all of stratified categories except localized PCa (HR = 1.10; 95% CI 0.93–1.31, *P* = 0.274), surgery (HR = 1.12; 95% CI 0.99–1.26, *P* = 0.072), radiotherapy (HR = 1.10; 95% CI 0.95–1.27, *P* = 0.198) for OS; and surgery (HR = 1.64; 95% CI 0.51–5.26, *P* = 0.404), prospective study design for PFS (HR = 1.42; 95% CI 0.89–2.26, *P* = 0.144) (Table 4). The high PLR also predicted poor OS in almost all of stratified categories except PCa (HR = 2.01; 95% CI 1.42–2.87, *P* = 0.068) and mCRPC (HR = 1.41; 95% CI 0.98–2.04, *P* = 0.068) (Table 5).

**Table 4:** Subgroup analyses of NLR for OS and PFS

	OS						PFS					*P*_H_
	No.	HR	95% CI	*P*	I-squared	*P*_H_	No.	HR	95% CI	*P*	I-squared	
Ethnicity												
Asian	10	1.83	1.52–2.21	0.000	0.0%	0.715	5	1.59	1.23–2.06	0.000	0.0%	0.436
Other	16	1.45	1.26–1.66	0.000	86.6%	0.000	7	1.62	1.18–2.23	0.003	79.7%	0.000
Patients status												
mCRPC	13	1.70	1.52–1.90	0.000	17.7%	0.265	6	1.62	1.33–1.98	0.000	45.3%	0.140
CRPC	5	1.86	1.40–2.48	0.000	0.0%	0.716	4	1.76	1.18–2.62	0.006	28.5%	0.221
Localized PCa	3	1.10	0.93–1.31	0.274	71.7%	0.029	1	3.09	1.64–5.82	0.000	–	–
All PCa	5	1.20	1.02–1.41	0.026	53.5%	0.072	1	0.84	0.74–1.19	0.603	–	–
Sample size												
≤ 300	15	1.73	1.40–2.16	0.002	59.6%	0.598	9	1.51	1.13–2.01	0.005	58.7%	0.013
> 300	11	1.45	1.23–1.71	0.000	89.5%	0.000	3	1.88	1.32–2.67	0.000	73.5%	0.023
Follow time												
≤ 30	12	1.74	1.55–1.95	0.000	0.0%	0.748	7	1.77	1.39–2.27	0.000	45.3%	0.089
> 30	7	1.15	1.02–1.30	0.020	75.2%	0.000	3	1.59	0.77–3.28	0.212	84.8%	0.001
Unclear	7	1.58	1.39–1.80	0.000	0.0%	0.430	2	1.36	1.00–1.88	0.061	0.0%	0.700
Cut-off												
≤ 3	10	1.75	1.57–1.97	0.000	0.0%	0.014	5	1.81	1.36–2.42	0.000	51.2%	0.085
> 3	12	1.63	1.32–2.00	0.000	53.8%	0.444	7	1.50	1.09–2.07	0.014	68.5%	0.004
No	4	1.14	0.97–1.34	0.101	88.5%	0.000	0	–	–	–	–	–
Study design												
Retrospective	21	1.63	1.39–1.91	0.000	84.0%	0.000	9	1.74	1.42–2.13	0.000	25.3%	0.219
Prospective	5	1.36	1.10–1.68	0.005	66.7%	0.017	3	1.42	0.89–2.26	0.144	84.3%	0.002
Statistical method												
Multivariable	22	1.54	1.34–1.77	0.000	79.5%	0.000	10	1.65	1.22–2.24	0.001	66.3%	0.002
Univariable	4	1.56	1.17–2.10	0.003	85.9%	0.000	2	1.66	1.20–2.30	0.002	74.1%	0.049
Therapy												
Surgery	5	1.12	0.99–1.26	0.072	72.7%	0.005	2	1.64	0.51–5.26	0.404	91.6%	0.001
Chemotherapy	18	1.69	1.53–1.84	0.000	1.2%	0.440	10	1.64	1.38–1.95	0.000	27.8%	0.188
Radiotherapy	1	1.10	0.95–1.27	0.198	–	–	–	–	–	–	–	–
Others	2	1.63	1.07–2.47	0.022	0.0%	0.638	–	–	–	–	–	–

OS, overall survival; PFS, progression-free survival; NLR, neutrophil to lymphocyte ratios; mCRPC, metastatic castration-resistant prostate cancer; PCa, prostate cancer; HR, hazard ratio; CI, confidence intervals; *P*_H_, heterogeneity of *P*-value

**Table 5:** Subgroup analyses of PLR for OS

	No.	HR	95% CI	*P*	I-squared	*P*_*H*_
Ethnicity						
Asian	3	2.01	1.42–2.87	0.000	0.0%	0.623
Other	3	1.52	1.11–2.09	0.009	0.0%	0.435
Patients status						
PCa	4	2.01	1.42–2.87	0.068	0.0%	0.623
mCRPC	1	1.41	0.98–2.04	0.068	–	–
Localized PCa	1	1.87	1.02–3.42	0.043	–	–
Sample size						
≤ 300	4	1.70	1.32–2.19	0.000	0.0%	0.421
> 300	2	1.87	1.02–3.42	0.043	–	–
Follow time						
≤ 30	2	1.49	1.06–2.10	0.022	0.0%	0.425
> 30	4	1.96	1.42–2.71	0.000	0.0%	0.615
Cut–off						
≤ 150	2	2.01	1.42–2.87	0.000	0.0%	0.655
> 150	3	1.52	1.11–2.09	0.009	25.7%	0.261
No	1	1.00	1.36–2.18	0.000	–	–
Statistical method						
Multivariable	3	1.75	1.16–2.62	0.007	0.0%	0.763
Univariable	3	1.78	1.23–2.58	0.002	28.7%	0.246
Therapy						
Surgery	2	1.87	1.02–3.42	0.043	–	–
Chemotherapy	2	1.48	1.09–2.01	0.012	0.0%	0.640
Radiotherapy	2	2.33	1.46–3.69	0.000	0.0%	0.798

OS, overall survival; PLR, platelet to lymphocyte ratio; mCRPC, metastatic castration-resistant prostate cancer; PCa, prostate cancer; HR, hazard ratio; CI, confidence intervals; *P*_H_, heterogeneity of *P*-value

## Discussion

Our present study, for the first time, included 32 studies to comprehensively evaluate the prognostic values of pretreatment systemic inflammatory factors (neutrophil, lymphocyte, platelet and monocyte counts as well as NRL, PLR and LMR) in patients with PCa. The results indicated that a high pretreatment NLR, PLR, neutrophil and monocyte counts predicted inferior OS outcomes; while elevated pretreatment LMR was correlated with favorable OS. In addition, the pooled data provided evidence that the higher NLR and monocyte counts, but lower LMR predicted worse PFS; poor RFS was only associated with pretreatment NLR. The subgroup analysis showed that the higher NLR may be a risk factor for prediction of OS only in patients with mCRPC and undergoing chemotherapy, but for PFS in patients with each status and chemotherapy; however, the higher PLR was only significantly associated with OS in localized PCa, but not mCRPC no matter of which therapy strategy.

Compared with the study of Yin et al. [20] (26 vs 14) and Cao et al. (26 vs 21) [21] which investigated the prognostic value of NLR for PCa, our study included more recently published literatures (13, 2016–2018) [12, 14, 18, 29–36, 38]. Also, several articles included in Yin et al. [20] and Cao et al. [21] were excluded because of inconsistent detection method (dNLR, the absolute neutrophil count divided by the difference between white cell and neutrophil counts; not NLR, the absolute neutrophil count divided by the absolute lymphocyte count) [53] or HR (95% CI) crudely extracted from the figures by software which may be inaccurate [54–58]. Thus, our conclusion may be more credible. Although our results seemed not completely similar to other two meta-analysis articles, they all indicated elevated pretreatment NLR was a prognostic predictor of shorter OS in mCRPC, a metastatic PCa. A high NLR indicated the number of neutrophils may increase. There have several studies to demonstrate neutrophilia promoted the metastasis of tumor cells [59, 60]. For example, Donati et al. reported neutrophil counts were elevated in premetastatic lungs in a syngenic mouse model using 4T1 tumor cells. Neutrophils may promote 4T1 cell adhesiveness, invasiveness, and migration by secreting cytokine IL-16, while instillation of an IL-16 neutralizing antibody reversed the effects of neutrophil on tumor cells [59]. Lu et al. proved the decreased release of C-X-C motif chemokine ligand 8, C-C motif chemokine ligand 2 (CCL2), CCL4, and matrix metalloproteinase-9 in neutrophils hampered the migration and invasion of oral squamous cell carcinoma cells [61]. The study of Wculek et al. supported that neutrophil-derived leukotrienes aided the colonization of distant tissue by selectively expanding the sub-pool of cancer cells that retain high tumorigenic potential. Genetic or pharmacologic inhibition of the leukotriene-generating enzyme arachidonate 5-lipoxygenase abrogated the pro-metastatic activity of neutrophils and consequently reduced metastasis [62]. Furthermore, there was also evidence to show neutrophilia facilitates the establishment of metastases by suppressing the anti-tumor activity of IL-17-producing γδ T lymphocytes [63]. Patients with a higher metastatic potential are more prone to die. In line with these studies, we also found pretreatment neutrophilia was an excellent predictor for poor OS.

Compared with the study of Wang et al. [22] on PLR, we included another two recently published literatures [17, 30] to expand the study samples (2994 vs 997). As expected, the results were inconsistent and the positive association between elevated PLR and shorter CSS demonstrated by Wang et al. [22] was not significant in our study. Only the relationship between a high pretreatment PLR and poor OS remained to be significantly demonstrated in both meta-analyses. Furthermore, we, for the first time, performed the subgroup analysis for PLR and the finding implied the higher PLR was only significantly associated with OS in localized PCa regardless of treatment options, but not mCRPC, suggesting PLR may be a prognosis factor for early stage. This conclusion seemed to be in accordance with the tumor proliferation-promoting roles of increased platelets by releasing platelet-derived growth factor and transforming growth factor [64].

Monocytes may reflect the formation of tumor-associated macrophages (TAMs). Extensive studies have suggested that monocytes/TAMs exert a pro-tumorigenic effect by the secretion of chitinase-3-like 1 (CHI3L1, YKL-40), interleukin-13 receptorα2 (IL-13Rα2) and then facilitating tumor cell invasion and migration [65, 66]. Thus, monocyte counts have been believed as a clinical prognostic factor for various cancers (including PCa), showing higher infiltration of monocytes predicts poor overall survival [14]. The higher monocytes counts may lead to the lower LMR and thus decreased LMR may be correlated with poor OS (in contrast, high LMR predict more favorable outcomes). This hypothesis was demonstrated in our study and several studies of other cancers [67, 68].

This meta-analysis has several limitations that should be acknowledged. First, the majority of eligible studies are retrospective which may carry a greater risk of selection bias. Second, the samples size is still not large. Only articles published in English language were included which may miss some studies with negative results published in native language. There were only 6 studies to be included for subgroup analysis of PLR and only two or three datasets enrolled to evaluate the prognostic significance of LMR and monocytes counts, which may result in the overestimation or underestimation of their prognostic values. Third, these inflammatory indicators are easily affected by the patients’ basic state such as age, tumor size, histological type, infection, and other inflammatory diseases. Some eligible studies did not describe these in detail that may cause the presence of heterogeneity. Fourth, inflammatory markers were analyzed in isolation of other prognostic markers. Further studies are needed to see their information value and (possible) influence on therapy in combination with clinical and molecular data. Fifth, all the inflammatory biomarkers were independently studied in the included articles. Thus, which or which combination may be more effective still cannot be confirmed in our study. Sixth, the NLR and PLR cutoff values used in the included studies ranged from 1.73 to 5 for NLR and 117.58 to 190 for PLR. This heterogeneity could hinder the application of these ratios in the clinical setting.

## Conclusion

Our meta-analysis preliminarily draw a conclusion that pretreatment elevated blood-based NLR, PLR, neutrophil and monocyte counts, but lower LMR are associated with worse OS in PCa patients. Detection of NLR, monocyte counts, and LMR may represent an inexpensive and easily available method for recurrence and progression prediction and may provide important supporting information to inform treatment decisions and predict potential treatment outcomes. The higher NLR and PLR may be a predominant risk factor for prognosis prediction for patients with mCRPC and localized PCa, respectively.

## Abbreviations

PCa: prostate cancer
OS: overall survival
TNM: tumor, node, metastasis
PSA: prostate-specific antigen
PLT: platelet
NLR: neutrophil–lymphocyte ratio
dNLR: derived NLR
PLR: platelet–lymphocyte ratio
LMR: lymphocyte–monocyte ratio
PFS: progression-free survival
CSS: cancer-specific survival
DMFS: distant metastases-free survival
RFS: recurrence-free survival
PRISMA: Preferred Reporting Items for Systematic Review and Meta-Analysis
HR: hazard ratio
CI: confidence interval
NOS: Newcastle–Ottawa Scale
CCL2: C-C motif chemokine ligand 2
CHI3L1: chitinase-3-like 1
IL-13Rα2: interleukin-13 receptorα2
TAMs: tumor-associated macrophages
